# Supplementary material for: Interplay between children’s cognitive profiles and within-school social interactions is nuanced and differs across ages
Source: Commun Psychol. 2025 Mar 20;3:44. doi: 10.1038/s44271-025-00227-4 (PMC11926095; doi:10.1038/s44271-025-00227-4)
Supplement: Supplementary file 2 — Supplementary material [file 44271_2025_227_MOESM2_ESM.pdf]

Supplementary Material

February 25, 2025

1 Cognitive test

Children were asked to take 8 or 9 tasks, depending on age. These are:

Execution Speed

Measures the speed at which a child could manipulate the test device, a valuable metric for evaluating timed tasks. The tasks consist of a four by five grids populated by a mole that changes position when clicked. The task consists of clicking on the mole as fast as possible. This task is timed and lasts for 30 seconds.

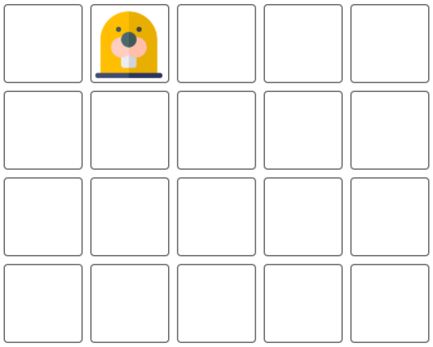

Figure S1: Screenshot for the Execution Speed task.

Reading Fluency

Measures the child's ability to quickly read and understand short sentences, focusing on decoding speed. The child is required to select the word missing in the sentence, from 5 choices. The task lasts for 180 seconds.

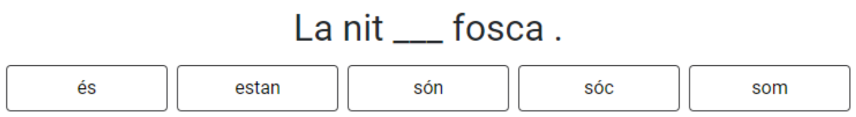

Figure S2: Screenshot for the Reading task. Note: the sentence reads ”The night ___ dark”, and the options are subtle Catalan variations of the verb ”to be” that can’t be faithfully translated.

Working Memory

This task evaluates the child’s ability to hold and manipulate information in the face of distractions. The child is shown a picture (figure A below) and asked to memorize it. Then, she has a task where she has to choose the odd one out among 4 drawings (figure B below). This is done 8 times with 8 different stimulus to remember and tasks to perform. Afterward, she is shown a grid with 24 images (figure C below) and has to pick as many as possible of the 8 pictures she has tried to memorize. This task is not timed.

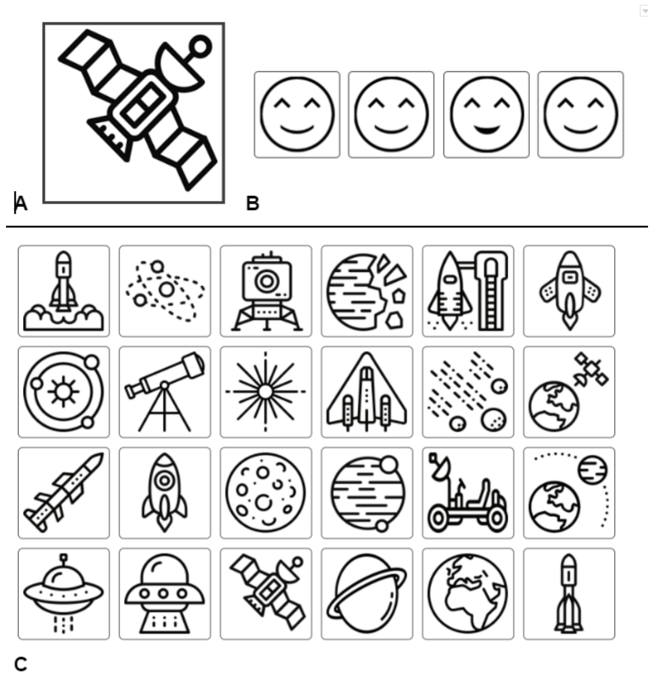

Figure S3: Screenshot for the Working Memory task.

Visual Processing Speed

This gauged the child's ability to perceive and make decisions based on simple visual stimuli quickly. The tasks consist in checking whether the upper symbol is repeated below, If it is, the child needs to click it, otherwise, she clicks "NO". This task is timed and lasts for 120 seconds.

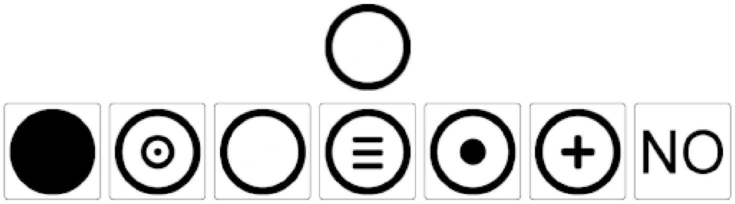

Figure S4: Screenshot for the Visual Processing Speed task.

Arithmetic Fluency

This measured a child's proficiency in performing rapid addition and subtraction within the 0-20 range. The child needs to do as many operations as possible in 120 seconds. Note that we designed a numerical entry object that looks like a normal calculator but holds the numbers from 0 to 19 so that students don't need to enter two separate digits when the result is over 9.

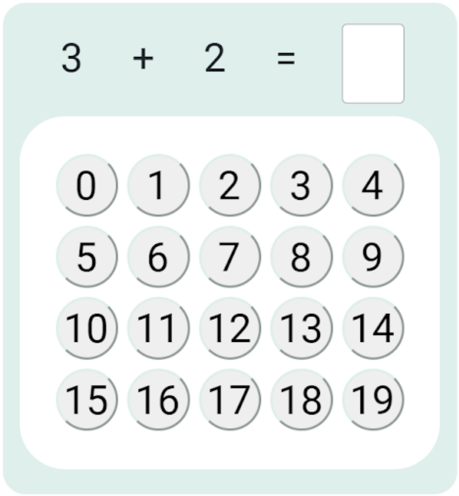

Figure S5: Screenshot for the Arithmetic Fluency task.

Long-Term Memory

This assessed the child's capability to retain information for periods longer than two minutes. The child is required to recall the same images that she memorized

in the Working Memory task. Te Long-Term Memory task is given to students around 10 minutes after the WM task. This task is not timed.

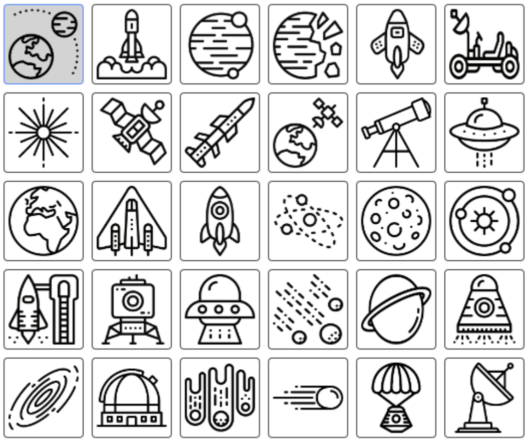

Figure S6: Screenshot for the Long-Term Memory task.

Reasoning

This task evaluates the ability to reason with concepts, infer logical patterns, and deduce their continuity. Children need to choose the element following the logical pattern from 4 options given below. This task lasts for 180 seconds.

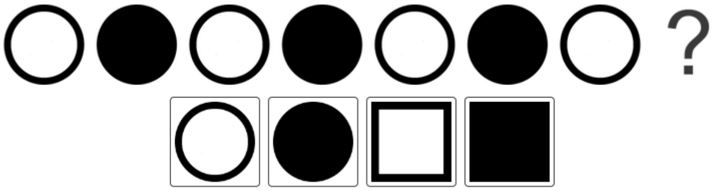

Figure S7: Screenshot for the Reasoning task.

Numerical Reasoning

This task, specific to children in grades 5 or above, gauged their capacity to handle more complex mathematical tasks beyond basic arithmetic. The child is required to fill either 1, 2, or 3 numbers or symbols missing in a mathematical equality. The task lasts for 180 seconds.

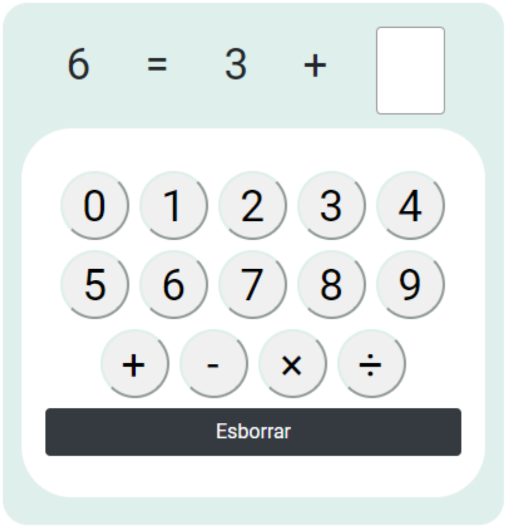

Figure S8: Screenshot for the Numerical Reasoning task.

Emotion Recognition

This measured a child's proficiency in recognizing emotions via facial expressions. In it, the child is required to select the emotion that the face is displaying. Options are "Happiness", "Fear", "Rage", "Surprise", "Sadness", and "No emotion", and the question reads "Which emotion does it represent?".

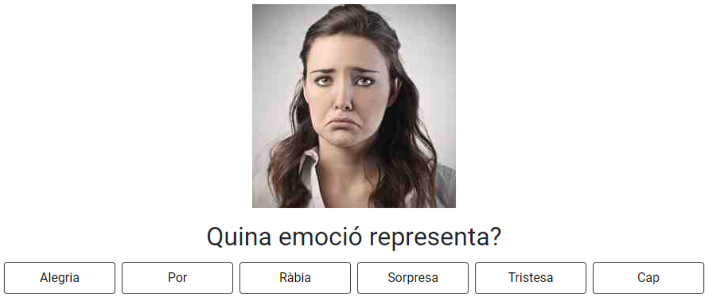

Figure S9: Screenshot for the Emotional Recognition task.

2 Sociogram

To understand children's social preferences and relationships within classroom settings, we incorporated sociometric techniques, which have been a cornerstone

in developmental psychology and education for their capability to decipher the intricate web of interpersonal preferences and antipathies among children [1, 2].

Assessment Design

The sociometric assessment employed a two-pronged approach:

1. **Indirect Questions**: Children were asked to nominate peers who fit specific descriptions across four domains:
   - Behavior: e.g., "Who is most likely to help if someone drops their books?"
   - Victimization: e.g., "Who gets teased or left out the most?"
   - Self-image: e.g., "Who is most confident in answering questions in class?"
   - Character: e.g., "Who is always honest, even if they get in trouble?"
2. **Direct Questions**: These were more straightforward, focusing on children's explicit preferences. They were instructed to name up to three classmates answering the following questions:
   - Who would you like to join your group to do an assignment?
   - Who would you NOT like to join your group to do an assignment?
   - Who would you like to join your group to play in the playground?
   - Who would you NOT like to join your group to play in the playground?
   - Who are you more friends with?

For the scope of this study, we concentrated on the responses to the direct questions. This focus allowed us to construct a nuanced snapshot of the social networks within each classroom, which has been shown to have profound implications on the socio-emotional and academic trajectories of students [3, 4].

3 Procedure for finding the optimal number of clusters for the achievement data

To determine the optimal cluster numbers, balancing model complexity and predictive accuracy, we employed the Tree-structured Parzen Estimator (TPE) algorithm, an advanced method for hyperparameter optimization [1]. Using the TPE, which adaptively focuses on promising hyperparameter regions, we analyzed an integer domain from 1 to 30, aiming to optimize the predictive accuracy of the MMSBM model [2,3]. The TPE analysis converged on three as the optimal cluster number for both tasks and student groups. This was based on maximized predictive accuracy relative to other counts and adherence

to the parsimony principle [4]. This relationship between cluster number and predictive accuracy is depicted in Fig. S11.

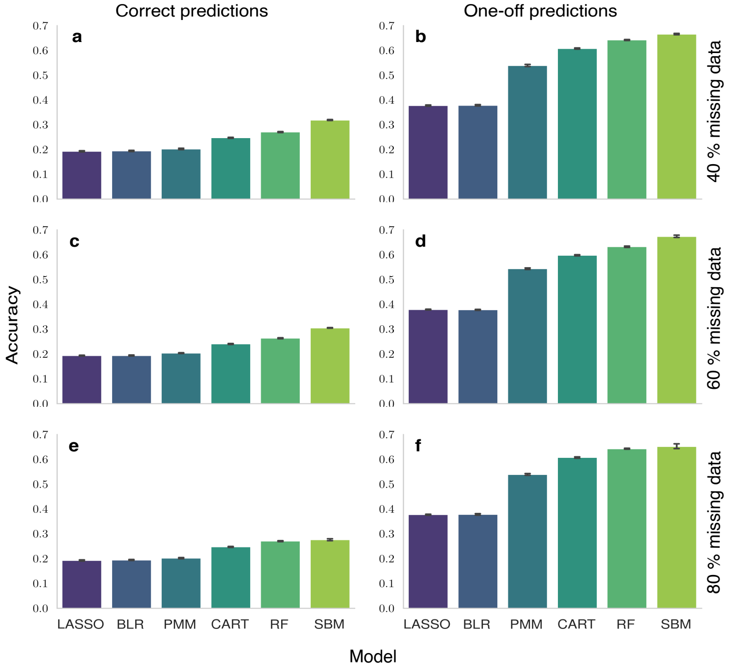

Figure S10: **Mixed Membership Stochastic Block Models prediction compared to other algorithms.** To assess the prediction power of MMSBM when compared to other state-of-the-art models, we perform predictive experiments in which we train the model on a partial observation of the data and make predictions over the missing data: 40% , 60%, 80%. For each prediction experiment, we show two types of performance metric: accuracy, that is the fraction of correct quintile predictions, and one-off-accuracy, i.e., the fractions of predicted quintiles that are at most one off the correct one . We show results for five different models (see Methods for details): Lasso linear regression (LASSO), Bayesian Linear Regression (BLR), Predictive Means Matching (PMM), Classification And Regression Trees (CART), Random Forests (RF) and the mixed-membership stochastic block model (MMSBM). Bars show the average over 20 experiments. Error bars show the standard deviation of the mean. Note that the one-off prediction models are also included in the main manuscript.

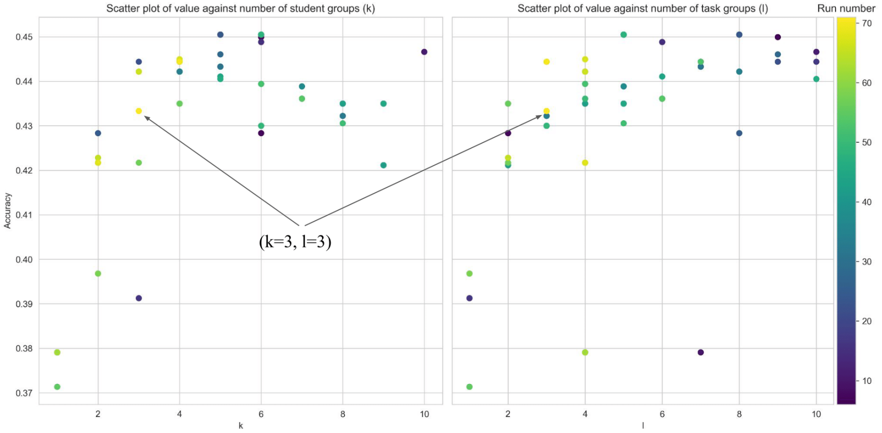

Figure S11: Accuracy, depending on the number of clusters, both for the cognitive profiles of children and for the tasks in the cognitive test.

4 Supplementary figures

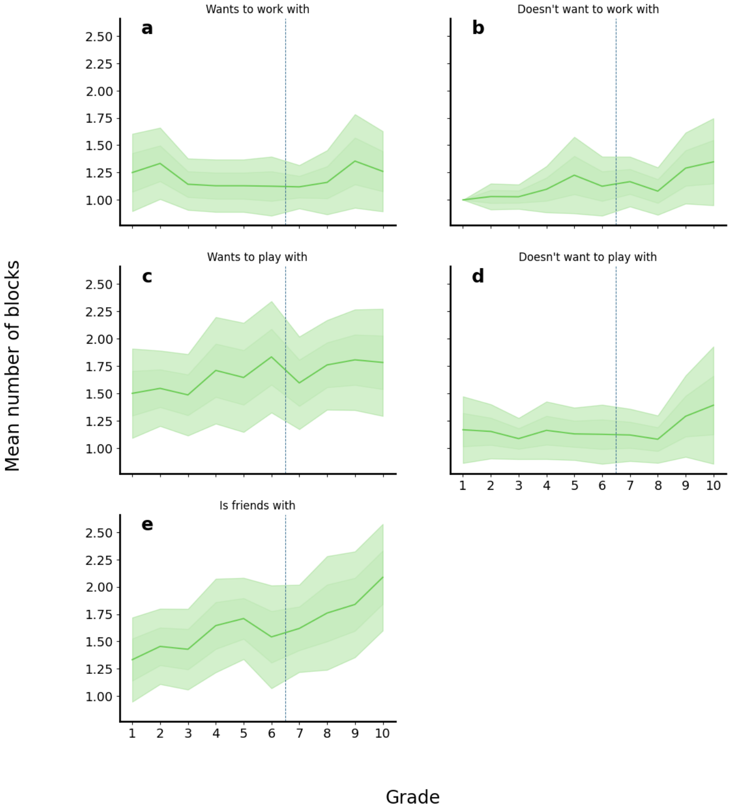

Figure S12: **Number of blocks per grade and per sociogram question.**

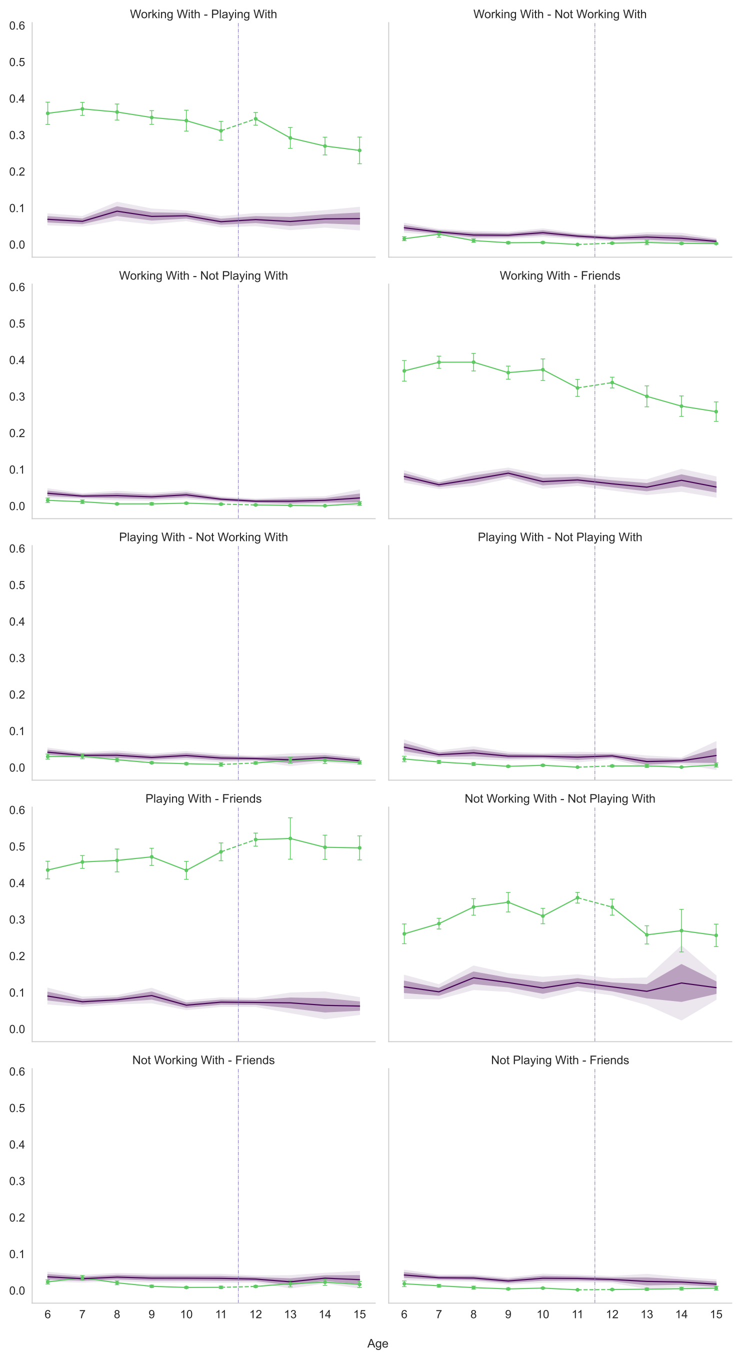

Figure S13: **Jaccard index of the overlap between all five layers of the sociogram.**

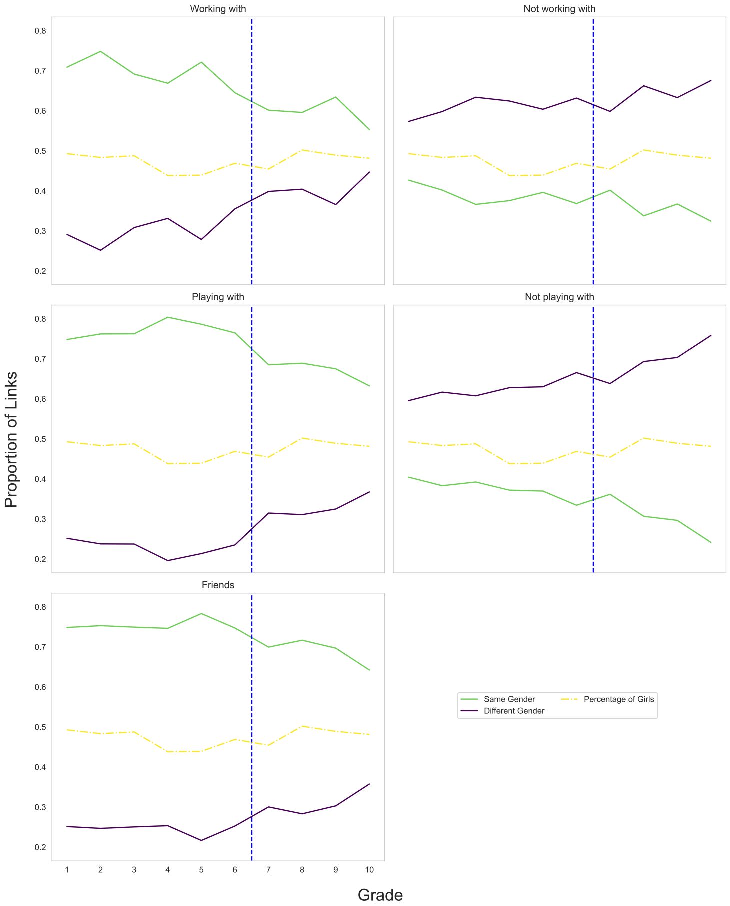

Figure S14: **Percentage of same gender choices (green) and different gender choices (purple) for each sociogram question.**

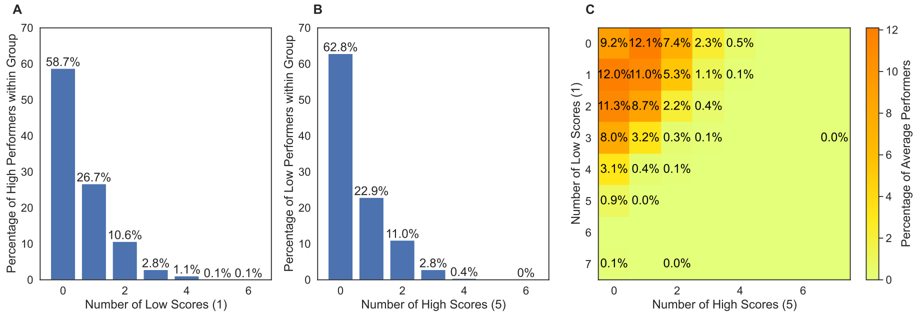

Figure S15: Distribution of tasks with extreme values (1 for the lowest quintile or 5 for the highest) of high, low, and average performing students. In A, we find the percentage of high performing students that have "n" tasks (in the x axis) in the lowest quintile. We can see that 58.7% of high performing students have no tasks at the lowest percentile (compared to the 0% for low performing and the 31.5% for the average performing students). In B, we find the distribution of low performing students that have "n" tasks (in the x axis) in the highest quintile. 62.8% of the low performing students have no tasks in the highest quintile, compared to the 1.1% of the high performers and the 44.6% for the average performing students. In C, we find the distribution of extreme values for the average performing students, where we observed that the majority have up to 3 lowest scoring tasks and up to 2 highest scoring tasks.

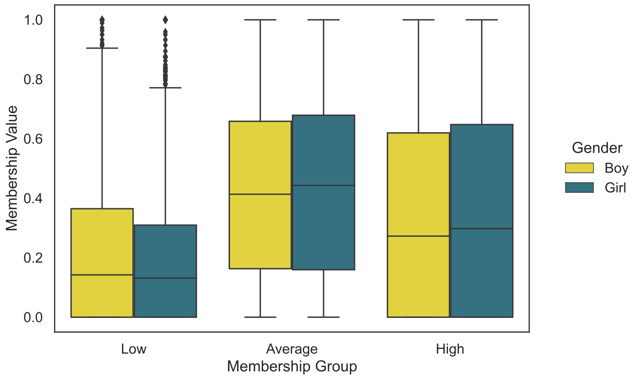

Figure S16: Distribution of group memberships per gender. When comparing the membership to the low, average, and high membership groups using two-sided Mann Whitney U tests, we find that only the membership to the low group is significant, with an effect size of 0,10 measured by Cohen's d, and p ¡ 0,001.

References

[1] Moreno, J. L. (1934). *Who shall survive? A new approach to the problem of human interrelations.* Washington, DC: Nervous and Mental Disease

Publishing Co.

[2] Coie, J. D., Dodge, K. A., & Coppotelli, H. (1982). Dimensions and types of social status: A cross-age perspective. *Developmental Psychology, 18*(4), 557-570.

[3] Bagwell, C. L., & Schmidt, M. E. (2011). The friendship quality of overtly and relationally victimized children. *Merrill-Palmer Quarterly, 57*(2), 158-185.

[4] Wentzel, K. R., & Caldwell, K. (1997). Friendships, peer acceptance, and group membership: Relations to academic achievement in middle school. *Child Development, 68*(6), 1198-1209.
